# Cranial ultrasound: and the risk of tunnel vision?

**DOI:** 10.1186/1824-7288-41-S2-A15

**Published:** 2015-09-30

**Authors:** Alberto Chiara, Stefania Perrini

**Affiliations:** 1 Paediatrics Department, Azienda Ospedaliera della Provincia di Pavia, Voghera and Broni-Stradella Hospitals, Italy

## 

“One man's one eyed perspective”

H.H. Fudenberg

The cranial ultrasound plays an important role in the study of the brain in the newborn and infant, therefore it represents the first choice technique to evaluate many diseases. In addition to the anterior fontanelle which serves as an acoustic window, the posterior fontanelle allows a more detailed study of the posterior fossa and occipital lobes [1,2]. Ultrasound is an irreplaceable, but not exclusive diagnostic tool; it is relatively simple to use in the majority of birth time points.

The ultrasound method has a high diagnostic value in the evaluation of hemorrhagic lesions, in ventriculomegaly, and in the form of cystic periventricular leukomalacia. Many lesions, however, are at risk of resulting as false positives for non-cystic periventricular leukomalacia. These are linked to the experience of the operator and the resolution of the equipment, as well as to white matter abnormalities. In these cases it is necessary to resort to magnetic resonance and, in particular, the new functional resonance techniques.

Neonatal hypoxic-ischemic encephalopathy, especially in the early stages, may result within normal range or exhibit only a diffuse hyperechoic picture, while more serious injuries may be recognized only later. Failure to visualize the lateral ventricles, the disappearance of the groove impressions and cerebral convolutions may be considered indirect signs of diffuse edema that may, however, be overestimated or underestimated [3].

**Figure 1:**
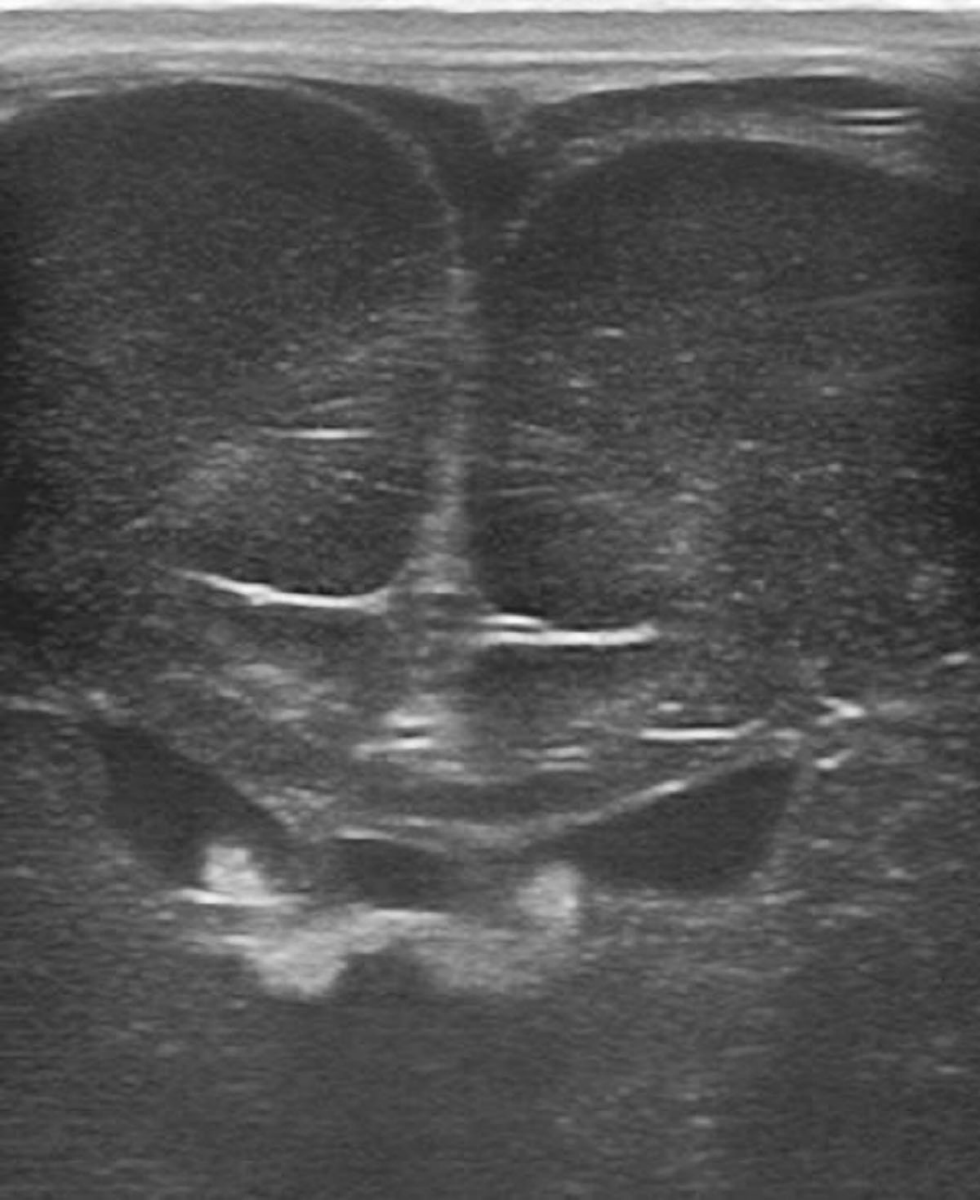
Infant with agyria (complete lissencephaly), the most serious form of abnormal neuronal migration. The coronal sonogram shows the complete absence of convolutions.

In congenital infections, ultrasound can easily identify some brain injuries, such as calcifications, germinolytic cysts or ventriculomegaly; while alterations of the posterior intracranial fossa are often underestimated [4], and although ultrasound is possible in disorders of neuronal migration they are inadequately diagnosed (Figure 1). In the case of neonatal meningitis - ultrasound can reveal complications such as ventriculitis, hydrocephalus and intraparenchymal abscesses, which fundamentally impact the choice of therapy.

Although technological advancements have permitted the production of economical ultrasound equipment with an acceptable level of quality, the gap in image quality and thus diagnostic power between top of the line and economical devices is generally abysmal. Currently, some ultrasound devices allow, under certain conditions, revelation of anatomical details that are almost invisible to the naked eye [5]. However, the use of proper methodology is fundamental, which necessitates the need for both accurate diagnostic and prognostic data, in order to avoid repetitive testing, and negative influences on future diagnostic - therapeutic decisions. It should be emphasized, however, that many ultrasound limitations are actually limitations associated with operator training and experience.
